# Supplementary material for: Imaging Erythrocyte Sedimentation in Whole Blood
Source: Front Physiol. 2022 Jan 28;12:729191. doi: 10.3389/fphys.2021.729191 (PMC8832033; doi:10.3389/fphys.2021.729191)
Supplement: Supplementary file 2 [file Image_1.pdf]

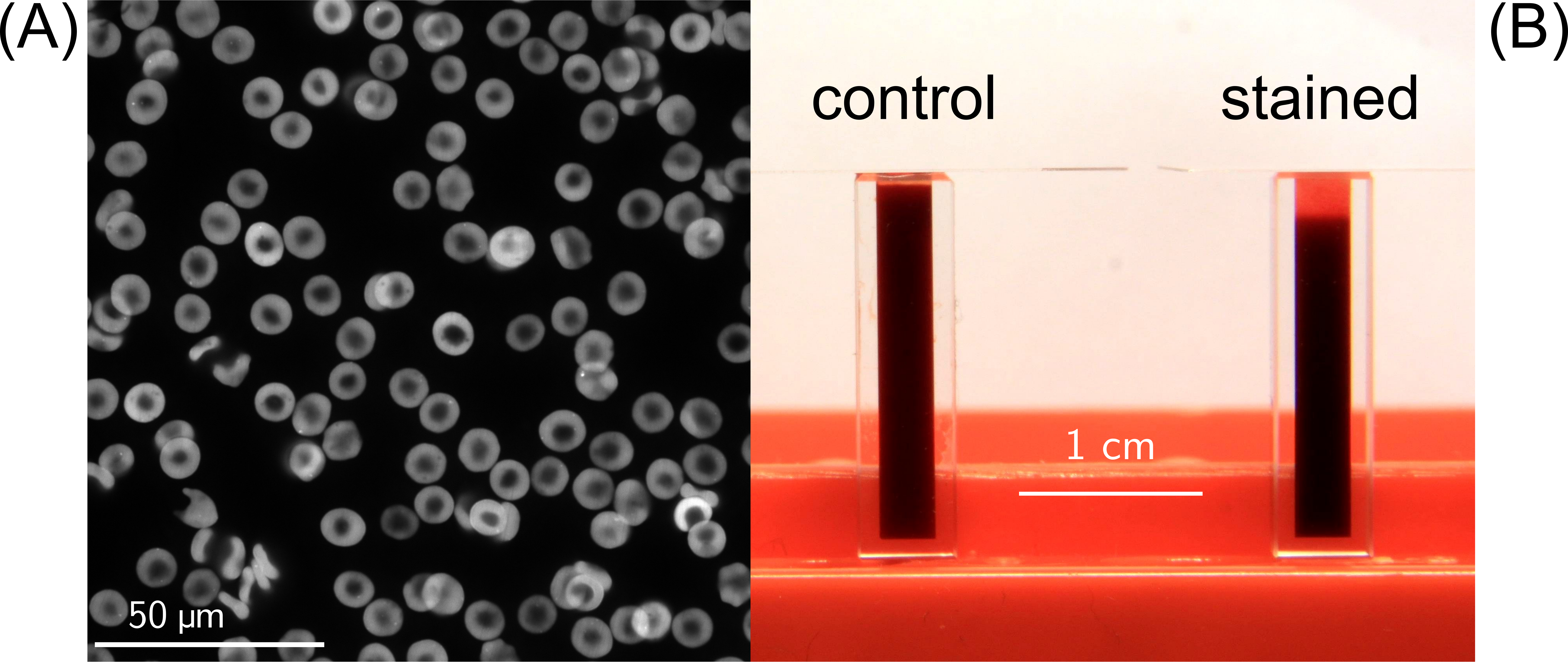

Supplemental Figure 1: Dyed cells behavior. (A) Representative photograph from dyed cells. Although the cells were stained correctly, the staining process modified their sedimentation behavior. (B) After two minutes at rest, the stained cells suspension (right container) presents a blurred interface with a reddish plasma on top. The control cells, at rest since 8 min, shows a smaller separation and a sharper interface. Both suspension have a controlled hematocrit of 45%. See also Movie 2 (same setup, sped up 420 times).
