# Supplementary material for: Imaging Erythrocyte Sedimentation in Whole Blood
Source: Front Physiol. 2022 Jan 28;12:729191. doi: 10.3389/fphys.2021.729191 (PMC8832033; doi:10.3389/fphys.2021.729191)
Supplement: Supplementary file 3 [file Image_2.pdf]

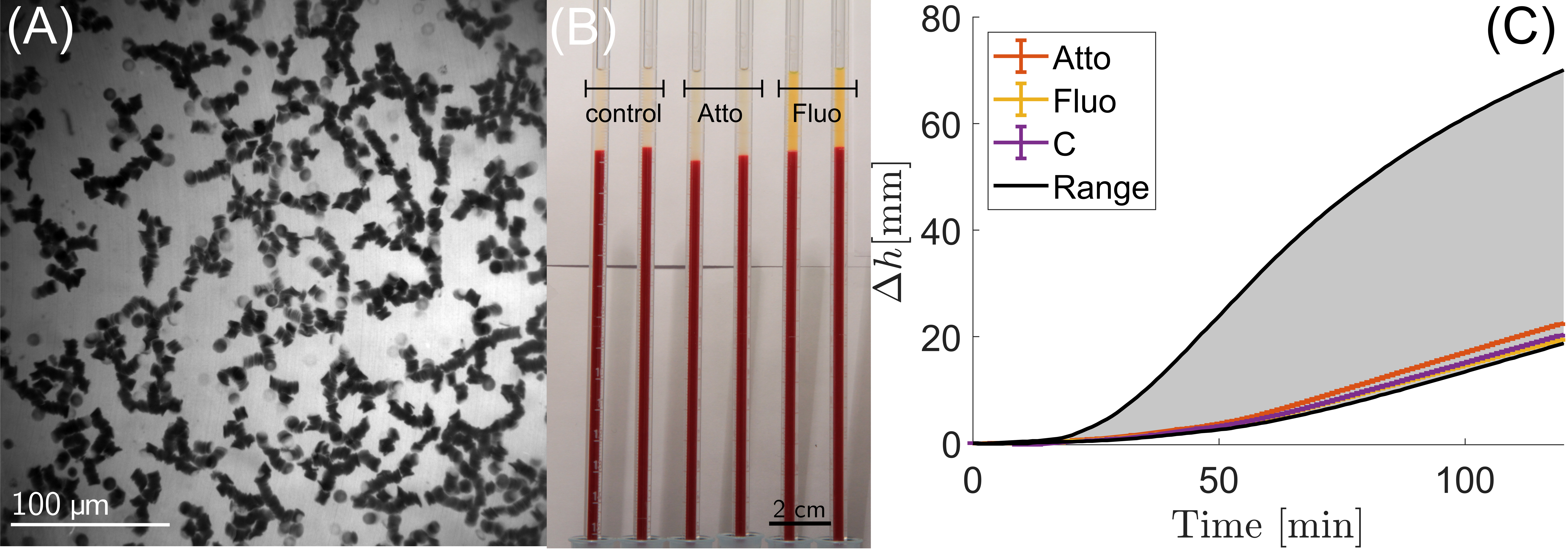

Supplemental Figure 2: Dyed plasma behavior. (A) Representative photograph from cells in dyed plasma. (B) After two hours at rest, the stained suspensions (with Atto647 (Atto) and Fluoresceine (Fluo)) presents a sedimentation rate with no significant difference from the control, unstained sample. All suspensions have a controlled hematocrit of 45%. (C) Shows usual range of sedimentation height along time for healthy samples, comprising the control measurements with the dyes.
