# Supplementary material for: Imaging Erythrocyte Sedimentation in Whole Blood
Source: Front Physiol. 2022 Jan 28;12:729191. doi: 10.3389/fphys.2021.729191 (PMC8832033; doi:10.3389/fphys.2021.729191)
Supplement: Supplementary file 4 [file Image_3.pdf]

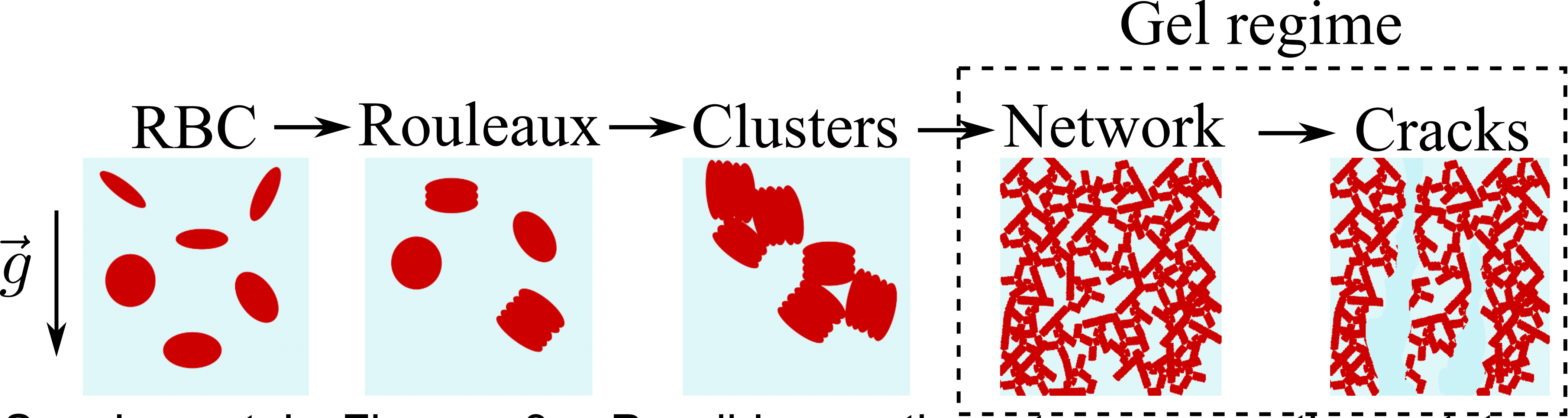

Supplemental Figure 3: Possible erythrocyte aggregation states. Dispersed erythrocytes (RBC) can aggregate into linear rouleaux, which can themselves assemble into larger, disordered aggregates. When those aggregates create a spanning network, they form what is called a colloidal gel. During this gel sedimentation, some cracks in the 3D structure usually appears. The last two stages define the gel regime of colloidal sedimentation.
